# Using Combined Computational Techniques to Predict the Glass Transition Temperatures of Aromatic Polybenzoxazines

**DOI:** 10.1371/journal.pone.0053367

**Published:** 2013-01-10

**Authors:** Phumzile Mhlanga, Wan Aminah Wan Hassan, Ian Hamerton, Brendan J. Howlin

**Affiliations:** Department of Chemistry, University of Surrey, Guildford, Surrey, United Kingdom; King's College London, United Kingdom

## Abstract

The Molecular Operating Environment software (MOE) is used to construct a series of benzoxazine monomers for which a variety of parameters relating to the structures (*e.g.* water accessible surface area, negative van der Waals surface area, hydrophobic volume and the sum of atomic polarizabilities, *etc.*) are obtained and quantitative structure property relationships (QSPR) models are formulated. Three QSPR models (formulated using up to 5 descriptors) are first used to make predictions for the initiator data set (*n* = 9) and compared to published thermal data; in all of the QSPR models there is a high level of agreement between the actual data and the predicted data (within 0.63–1.86 K of the entire dataset). The water accessible surface area is found to be the most important descriptor in the prediction of T_g_. Molecular modelling simulations of the benzoxazine polymer (minus initiator) carried out at the same time using the Materials Studio software suite provide an independent prediction of T_g_. Predicted T_g_ values from molecular modelling fall in the middle of the range of the experimentally determined T_g_ values, indicating that the structure of the network is influenced by the nature of the initiator used. Hence both techniques can provide predictions of glass transition temperatures and provide complementary data for polymer design.

## Introduction

**Figure 1:**
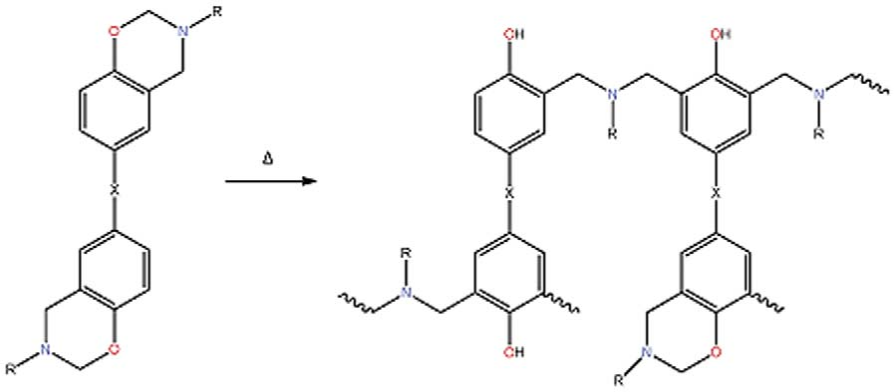
Polymerisation of bisbenzoxazines through ring opening and crosslinking.

Polybenzoxazines form a comparatively new family of thermosetting resins that are being explored as potential higher performance replacements for phenolic resins. The preparation of aromatic oxazines, or benzoxazines, date back some sixty years [1] but commercial polymers based on *bis*-benzoxazines are comparative newcomers to the scene, but have already justified the publication of a recent handbook [2]. Poly(*bis*-benzoxazine)s (sometimes simply referred to as polybenzoxazines) are formed through step growth ring-opening polyaddition from *bis*-benzoxazine monomers (Figure 1), which are in turn the products of the Mannich reaction between a *bis*-phenol, formaldehyde and a primary amine [3]. The monomer-oligomer ratio in the yield can also be influenced by using an excess of formaldehyde and amine during the synthesis; causing the products to form *via* a different mechanism and resulting in a greater proportion of monomer in the product [4]. This, in turn, affects the properties of the resin before, during and after cure (the presence of oligomers bearing hydroxyl groups in the chain is known to enhance the reactivity of the benzoxazine). Polybenzoxazines appear to incorporate the best properties from conventional phenolics, and may find application in a number of their traditional niches, whilst improving on shelf life and offering the potential for greater toughness properties through their greater molecular flexibility; the relative cheapness of the monomer is also an important factor influencing their adoption. Unlike many other commercial thermosetting resins, which evolve condensation products such as water or ammonia, benzoxazine monomers react relatively cleanly to form a polymer with few reaction by-products [5] although the exact manner of the polymerisation reaction to form a network has not been fully elucidated. The glass transition temperature is when the polymer goes from a glassy to a rubbery state. This is not a thermodynamic change of state so there is no exact value rather a range over which it occurs. Hence the experimentally determined value depends to a certain extent on how it is measured and quoted values can differ by plus or minus 10–20 K. There are a number of empirical equations to predict T_g_, the Fox equation, the Gordon and Taylor equation, the Kwei equation and first published in 2008, the equation of Brostow et al. [6], which uses a cubic polynomial based approach to predict the T_g_ values of polymer blends. The simulation of the thermal and mechanical properties of polymers is an area of growing interest. There are basically two main methods employed for this; the first of which is quantitative structure property relationships (QSPR) where group additive methods are used to derive values of the properties of interest. The second method is atomistic simulation, which uses full atomic detail of the polymers. The prediction of thermal and mechanical properties in as yet unsynthesised polymers is beginning to be realised and we have been demonstrating this by the second method in a variety of thermosetting polymers such as epoxy resins [7], cyanate esters [8] and polybenzoxazines [9], as well as engineering thermoplastics [10], [11]. The QSPR method was initially pioneered by Van Krevelen culminating in a book published in 2009 [12]. However, there have been a large number of approaches to QSPR in polymers, including force field approaches [13] and the inclusion of quantum chemical descriptors [14]–[16]. The CODESSA program has been used to develop an empirical equation for predicting the T_g_ values of high molecular weight polymers using five descriptors related to shape and intermolecular electrostatic interactions [14]. The Dow chemical company has been very active in this area and have combined QSPR in the SYNTHIA program with Accelrys software to make a capability for high throughput reverse engineering design of new polymer chemical structures [17]–[19]. Other approaches have involved the use of neural networks [20], [21] and the EVM Method [22]. The EVM method [22] uses energy, volume and mass to predict the Tg values of acrylate and methacrylate polymers with an error of no more than 10%. In a previous publication [23], we reported the use of a quantitative structure property relationship (QSPR) to predict the Tg of a polymer of this type, but the model was severely limited by the size of the training set used to generate the QSPR equation. In our recent work, a much more extensive study was reported extending the approach to an extensive range of poly(aryl ether sulphone)s [24]. In this paper we present the results of a study into the thermal polymerisation of polybenzoxazines and develop a methodology to predict the glass transition temperatures of the cured polymers by two methods, one based on quantitative structure property relationships and one based on atomistic modelling. Both techniques are shown to be capable of predicting the glass transition temperatures to a good degree of accuracy and provide complementary information for the computer aided design of polymers of benzoxazines.

## Experimental

### Materials

CuCl_2_ (99.999%) and MnCl_2_ (99%) were purchased from Sigma Aldrich (St. Louis, USA). CuCl (97%), FeCl_3_ (99.98%) and AlCl_3_ (99%) were purchased from Alfa Caesar, (Massachusetts, USA). NiCl_2˙_6H_2_O (97%), ZnCl_2_ (98%) and FeCl_2˙_4H_2_O (99%) were purchased from BDH Prolabo Chemicals (Leicestershire, UK). The benzoxazine monomer (BA-a, Araldite MT35600) was obtained from Huntsman Advanced Materials (Basel, Switzerland).

### Polymerizations of BA-a with a variety of initiators

Incorporation of the various initiators with the monomer was achieved by heating BA-a to its melting point (58–70°C), maintaining the temperature and then adding the initiator while stirring using a magnetic stirrer bar. The molar ratio of monomer to initiator (20∶1) was kept consistent with that reported in the study conducted by Wang and Ishida [25]. Once a homogeneous blend had been achieved the mixture was quenched, allowed to cool, ground and stored at below 0°C prior to analysis.

### Apparatus

Differential scanning calorimetry was undertaken using a TA Instruments Q1000 on samples of 5±0.5 mg in hermetically-sealed aluminium pans. Experiments were conducted at a heating rate of 10 K min^−1^ from 20–300°C under flowing nitrogen (50 cm^3^ min^−1^). The samples were then cooled at 10 K min^−1^ from 300 to 20°C before a rescan was run at 10 K min^−1^ (25–300°C) to reveal glass transition temperature (T_g_) for samples (taken as the midpoint of the transition).

### Generation of QSPR Models

The Molecular operating Environment (MOE) software [26] was used for quantitative structure-property relationship (QSPR) modelling. MOE was used to generate models to calculate glass transition temperature (T_g_) for a single monomer following initiation with various known initiators and for various *bis*-benzoxazine monomers. The general procedure that was followed for each property of interest can be summarised in five stages: (i) the variable of interest (*i.e.* T_g_) was fitted to a range of independent variables (descriptors) within the database to generate a preliminary QSPR model. The process for selection of appropriate descriptors was broadly based on trial and error, with the criteria that a suitable QSPR model should incorporate as few descriptors as possible and display a correlation coefficient value (R^2^) greater than 0.99. (ii) Cross validation was achieved by making a comparison of the actual data used as the training set to the data predicted using the QSPR model, testing using Leave One Out Cross Validation (LOOCV) and calculation of the $XZ-SCORE cross validation property – where suitable. The $XZ-SCORE is defined as the absolute difference between the value of the model under a LOOCV scheme and activity field, divided by the square root of the mean square error of data [27]. (iii) The models were tested by generating data for new initiators/monomers and comparing the predictions made based on the QSPR models, to the experimental data. (iv) The descriptors were ‘pruned’ in order to select the optimum set. (v) The most significant or influential descriptors or sets of descriptors were identified for each property.

### Generation of Atomistic Models

The molecular modelling program Accelrys Materials Studio version 6.0 [28] was utilised within this work and all the modelling work was carried out using an in house PC (Dell Latitude E6520, Intel Core Duo 2.50 GHz, 4.09 GB RAM). The potential energy for all models throughout this work was calculated using the Condensed-phase Optimised Molecular Potential for Atomistic Simulation Studies (COMPASS) [29], a force field specifically designed for polymer calculations. To form the cross-linked polymer network (the polybenzoxazine of BA-a) to different selected degrees of conversion, a curing programme produced in house [30] was employed with the PCFF force field. For the construction of the network, the cut-off was set at between 5.0–6.0 Å, the dynamics duration was set at between 1,000 and 10,000 ps and the simulated cure temperature was 453 K (180°C). The network produced under these conditions was apparently 79% cured (α = 0.79), which is in good agreement with literature data for a post cured sample (*cf* α = 0.70 achieved after 15 minutes at 210°C [30]) suggesting that the cut off distance during the curing programme is not currently optimised and work continues to address this.

### Molecular Simulation After Cure

The temperature ramped Molecular Dynamics (MD) simulations were performed using the Temperature Cycle option in the Amorphous Cell Protocols. A collection of MD simulations was run over different temperatures, with decrements of 10 K from the starting temperature. The starting temperature was set at 573 K, and a typical total of 31 MD simulations were performed, ranging between 573 K and 273 K. At each temperature stage a 125 ps MD simulation was created. The first 25 ps of each simulation were used to equilibrate the system and the subsequent 100 ps simulation was used to record the results. The NPT ensemble (298 K, 0.0001 GPa) with a time step of 1 fs was utilized with the Anderson thermostat in combination with the Parinello Barostat [31]. COMPASS was used with the atomic van der Waals summation, a cut-off at 10.00 Å, a spline width of 3.00 Å and a buffer width of 1.00 Å.

The T_g_ is a second order phase change, which shows a change in thermal expansion coefficient when the temperature and volume of a polymer are plotted. The point of gradient change in the plot pinpoints the position of the T_g_. The process of indicating the best point of gradient change can be quite complex. Hall *et al.* [32] developed an in-house technique of calculating this hinge point by finding when the fit quality of a line is at its maximum. This method is based on finding the best fit for a gradient change as a function of temperature. As the degradation temperature of polymers shows a similar change in volume as a function of the temperature, the same technique has been applied within this work.

## Results and Discussion

### Generation of QSPR models to predict T_g_ for poly(BA-a) using different initiators

MOE 2011.10 has a database of 334 descriptors divided into three classes, 2D descriptors that only use atom and connection data; i3D that use 3D coordinate information but are invariant to rotations and translations of the molecule and ×3D that also use 3D coordinate information but require a frame of reference for the molecule set. In the initial stage of design of a QSAR equation one does not know which descriptors to choose, so an inspired guess is made. As we are trying to model T_g_, it was thought that size and shape would be important, so descriptors that related to these were chosen. Each iteration of model fitting gives an R^2^ value for the fit along with an evaluation of the relative importance of each descriptor to the fit. This relative importance is presented on a scale of 0–1, with 1 being the most important. Therefore after each iteration, the descriptor with the least importance was deleted and the fit recalculated until the R^2^ value was as close to 100% as possible with the set of descriptors chosen. New descriptors were then added to the set and the process repeated until the maximum R^2^ value with the minimum set of descriptors was found. Naturally it is impossible to try all combinations and with the small size of the datasets there will be several possible solutions anyway. Initially, the data presented in Table 1 (taken from reference [33] were used as a training set. Three QSPR models were deemed suitable for the prediction of T_g_ (Table 2); all had R^2^ values >0.99 indicating a reasonable degree of confidence and of these, QSPR model 1.2 had the highest R^2^ value (0.999), although all three were similar in magnitude.

**Table 1:** Comparison of T_g_ data for poly(BA-a) cured with selected initiators.

Initiator	Formula	T_g_ (°C)
Phosphorus pentachloride	PCl_5_	215
Phosphorus trichloride	PCl_3_	216
Phosphorus oxychloride	POCl_3_	210
Titanium(IV) chloride	TiCl_4_	222
Aluminium chloride	AlCl_3_	186
Methyl tosylate	C_8_H_10_O_3_S	142
Methyl triflate	C_2_H_3_F_3_O_3_S	193
Aluminium phthalocyanine chloride	C_32_H_16_AlClN_8_	186

**Table 2:** QSPR models to predict T_g_ for poly(BA-a) with selected initiators (molar equivalence of initiator = 20∶1).

QSPR model	r^2^	Estimated linear model	Weighting of descriptor
1.1	0.990	379.359−0.588(PEOE_VSA_HYD)−0.941(ASA)+0.865(vsurf_Wp3)+0.463(zagreb)	0.45 PEOE_VSA_HYD, 1.00 ASA, 0.12 vsurf_Wp3, 0.19 zagreb
1.2	0.999	374.953+17.513(dipole)+0.630(PEOE_VSA_HYD)−0.946(ASA)+1.085(vsurf_Wp3)+0.272(zagreb)	0.11 dipole, 0.47 PEOE_VSA_HYD, 1.00 ASA, 0.15 vsurf_Wp3, 0.11 zagreb
1.2	0.994	3360.462+24.124(dipole)−0.875(ASA)+0.629(PEOE_VSA_HYD)+1.093(vsurf_Wp3)	0.16 dipole, 1.00 ASA, 0.52 PEOE_VSA_HYD, 0.16 vsurf_Wp3

Key: ASA = water accessible surface area, PEOE_VSA_HYD = total hydrophobic van der Waals surface area, vsurf_Wp3 = polar volume at −1.0 Å, zagreb = zagreb index.

### Validation of QSPR model

The data set (*n* = 8) was deemed too small for the use of LOOCV for validation, but the $Z-SCORE cross validation properties were calculated for each QSPR model where a $Z-SCORE value of ≥2.5 indicates molecules that are outliers in the fit [26]. In all three of the QSPR models the $Z-SCORE values were all well below 2.3 indicating that there were no outliers in the data sets. The highest $Z-SCORE that was observed was 2.27 with POCl_3_ in QSPR model 1.2; a similar finding was reported for this initiator in a study focused on prediction of char yield in polybenzoxazines [34], [35]. The predicted T_g_ values of the polybenzoxazines formed from BA-a in the presence of the different initiators. The three QSPR models (1.1–1.3) were first used to make predictions for the initiator data set and compared to the actual data originally reported by Wang and Ishida [25]. The results are shown in Table 2: in all of the QSPR models there was a high level of agreement between the actual data and the predicted data. Based on comparisons of the predicted data generated for the different QSPR models, QSPR model 1.2 had the lowest average difference. Notably, the highest differences between the predicted data and actual data were found with POCl_3_ which also had the highest $Z-SCORE values in all the QSPR models.

### Analysis of QSPR model descriptors

In terms of the descriptors, the water accessible surface area (ASA) was the most important descriptor in the prediction of T_g_. The water accessible surface area is defined as the area over which the centre of a water molecule can be placed while retaining van der Waals contact with that atom and not penetrate any other atom [36]. The accessible surface area is inversely related to the molecular surface or Connolly surface [37] and provides a smoother interpretation of the surface of a molecule. Whilst it is true that T_g_ is measured in the bulk state, without the presence of solvent, the ASA effectively includes the excluded volume of the polymer chains, *i.e.* when the chains are in motion at a given temperature they cannot fit together exactly on van der Waals volume. This may give some support to the old united atom theory that was used in the past to model polymer chains. The ASA descriptor falls under the category of descriptors related to surface area, volume and shape, and is calculated by using a radius of 1.4 Å for the water molecule. A polyhedral representation is used for each atom when calculating the surface area [27]. The second most important descriptor in all three QSPR models was the total hydrophobic van der Waals surface area.

### Comparison of experimental data against predictions calculated using QSPR model

The QSPR model data were compared to data obtained experimentally in house. The following initiators (phosphorus pentachloride, phosphorus trichloride, phosphorus oxychloride, titianium(IV) chloride, aluminium chloride, methyl tosylate, methyl triflate and aluminium phthalocyanine chloride) were successfully incorporated into the BA-a monomer and analysed by DSC to determine T_g_. Thus, the QSPR models presented in Table 3 were applied to predict T_g_ for poly(BA-a) when cured with the new set of initiators. Seven transition metal chlorides (CuCl_2_, CuCl, MnCl_2_, NiCl_2_, ZnCl_2_, FeCl_2_ and FeCl_3_) were chosen for testing the QSPR models. Predictions were made and compared to observed experimental data. The predictions made based on QSPR model 1.1 are presented in Table 4.

**Table 3:** Comparison of T_g_ data for poly(BA-a) using selected initiators.

QSPR model	Initiator	Reported Tg (°C) [25]	Predicted Tg (°C)	Average difference (°C)
1.1	PCl_5_	215	216	1.86
1.1	PCl_3_	216	210	1.86
1.1	POCl_3_	210	212	1.86
1.1	TiCl_4_	222	222	1.86
1.1	AlCl_3_	186	189	1.86
1.1	Methyl triflate	193	193	1.86
1.1	Methyl tosylate	142	141	1.86
1.2	PCl_5_	215	215	0.63
1.2	PCl_3_	216	215	0.63
1.2	POCl_3_	210	212	0.63
1.2	TiCl_4_	222	223	0.63
1.2	AlCl_3_	186	186	0.63
1.2	Methyl triflate	193	192	0.63
1.2	Methyl tosylate	142	142	0.63
1.3	PCl_5_	215	214	1.63
1.3	PCl_3_	216	218	1.63
1.3	POCl_3_	210	212	1.63
1.3	TiCl_4_	222	222	1.63
1.3	AlCl_3_	186	187	1.63
1.3	Methyl triflate	193	189	1.63
1.3	Methyl tosylate	142	143	1.63

**Table 4:** QSPR models to predict T_g_ for bisphenol A polybenzoxazines (molar equivalence of initiator = 20∶1).

Initiator	Predicted T_g_ (°C)	μ±1 σ	μ±2 σ	μ±3 σ	Observed T_g_ range (°C)
CuCl_2_	194	194–195	193–196	193–196	119–218
CuCl	256	255–256	255–257	254–258	118–211
MnCl_2_	199	198–199	197–200	197–201	107–214
NiCl_2_	237	234–235	233–236	233–236	115–213
ZnCl_2_	198	198–199	197–200	196–200	114–220
FeCl_2_	234	234–235	233–236	232–236	118–216
FeCl_3_	250	250–251	249–252	249–252	111–213

T_g_ obtained by DSC rescan at 10 K/min.

The Gaussian distribution is used in statistical analysis to represent the frequency of distribution of experimental data. Based on the Gaussian distribution, 68% of the data should be within ± one standard deviation (σ) from the mean value. In this case, the standard deviation applied was based on the mean values obtained for the Wang and Ishida [25] initiator set, where the mean value (μ) was set as the predicted value for that particular initiator. One challenge is the assignment of T_g_, which is variously reported as the onset of the loss in storage modulus, the peak in the loss modulus, or the tan δ trace. More correctly, T_g_ commonly occurs over a range of temperature, rather than at a single temperature and thus our data are compared with the temperature range determined from DSC data.

### Molecular simulation of glass transition temperature for poly(BA-a)

**Figure 2:**
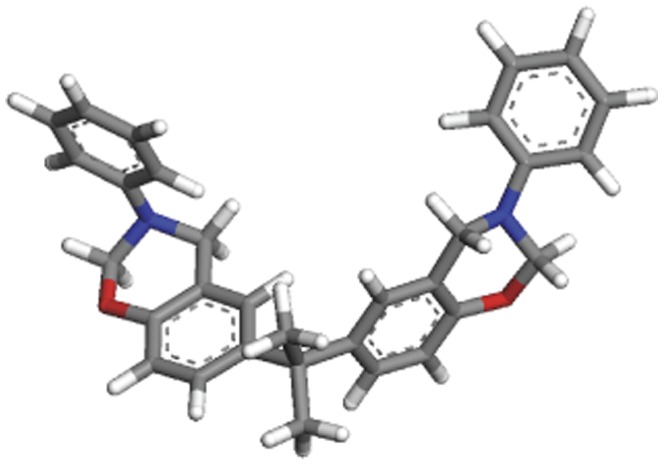
3D atomistic model of the BA-a monomer constructed using Materials Studio.

Prior to examining the glass transition temperature, it was necessary to examine the molecular mechanics data to determine whether the *bis*-benzoxazine monomer (BA-a) was well reproduced before embarking on a study of the corresponding polymer. The structure is shown in Figure 2, with the chair-like conformation of the benzoxazine rings, clearly visible. Furthermore, comparison of selected simulated bonds lengths and angles (following energy minimisation) with analytical data obtained using X Ray crystallography (Table S1) reveals the close agreement between the two, offering confidence in the subsequent simulation experiments involving the crosslinked forms of this material.

**Figure 3:**
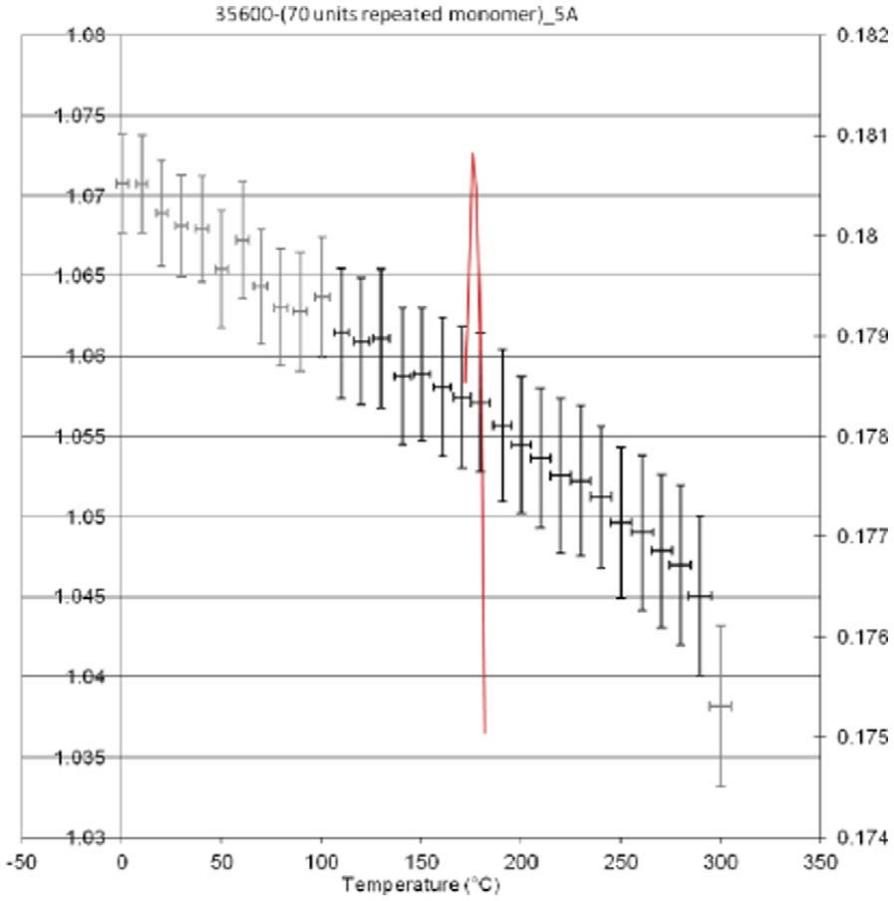
Plot of density (left axis) *versus* temperature of poly(BA-a) (cured using cut off distance of 5 Å).

**Figure 4:**
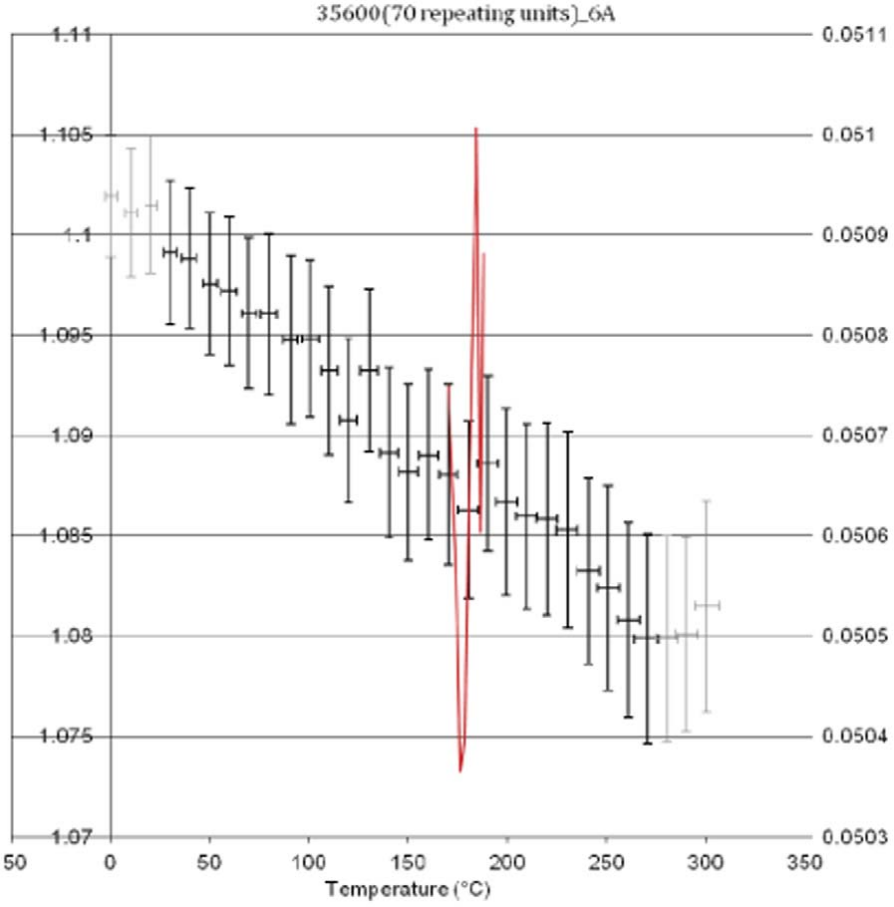
Plot of density (left axis) *versus* temperature of poly(BA-a) (cured using cut off distance of 6 Å).

Two project/simulations were completed for poly(BA-a) by setting the programme to cure within a 5 Å distance, which yielded a densely cured network (α = 0.79). The temperature cycle protocol was executed using the conditions in Table 4 and applied to all frames (Figure 3). Under these conditions the simulated T_g_ for poly(BA-a) is *ca.* 170°C (*cf* literature values of 170°C [38]).The drop in simulated polymer density is quite marked and perhaps reflects the empirical data reported for a similar polymer [39] in which DMTA measurements show a drop of some 1200 MPa between zero and 150°C. The simulated density is also in good agreement with the reported [25] empirical value for the poly (BA-a) of 1.195 g/cm^3^. From the same study, the glass transition temperature of the polybenzoxazine was determined as (*ca.* 169–173°C based on the peak maxima values of the loss modulus. In the present study, the line indicating the best fit in the simulation for the change in density is found to lie between *ca.* 170 and 180°C. A second simulation was undertaken using a longer cutoff distance (6 Å) during cure, which resulted in a significantly larger value for conversion (α = 0.88) and the initial density is slightly higher (1.102 g/cm^3^ *cf* 1.07 g/cm^3^) (Figure 4).

## Conclusions

QSPR techniques have been shown to be effective in predicting experimental T_g_ values for a data set based on a polybenzoxazine derived from bisphenol A and aniline when blended separately with eight well characterised initiators. Using model equations formulated involving up to five descriptors agreement is found to be good (within 0.63–1.86 K of the entire dataset). When the model is extended to a new data set including seven initiators, the reliability is reduced somewhat, but still able to able to predict the T_g_ values (±3 σ) within the empirical range for 6 of these. The QSPR model indicates the importance of the surface area of the initiator in influencing polymerisation and T_g_. The use of MD simulation (atomistic modelling for oligomers of 70 repeat units and a cut off distance of 6 Å) offers good agreement with the QSPR models and experimental values yielding T_g_ values within the middle of the empirical ranges. The two models can be used to predict the glass transition temperatures of polybenzoxazines, the QSPR providing information on the variation of T_g_ with respect to initiator used and the atomistic simulation providing information on the network resulting from the polymerisation.

## Supporting Information

Table S1 Selected bond distances and angles obtained for BA-a using Materials Studio (for the given conformations in Figure 1).
(DOC)
