# Supplementary material for: Using Combined Computational Techniques to Predict the Glass Transition Temperatures of Aromatic Polybenzoxazines
Source: PLoS One. 2013 Jan 10;8(1):e53367. doi: 10.1371/journal.pone.0053367 (PMC3542367; doi:10.1371/journal.pone.0053367)
Supplement: Table S1 — Selected bond distances and angles obtained for BA-a using Materials Studio (for the given conformations in Figure 1). (DOC) [file pone.0053367.s001.doc]

Table S1. Selected bond distances and angles obtained for BA-a using Materials Studio (for the given conformations in Figure 1).

|  | | | | | |
| --- | --- | --- | --- | --- | --- |
| **Bond lengths (Ǻ)** | | | **Bond angles (˚)** | | |
|  | **Modelling data** | **Empirical data^1^** |  | **Modelling data** | **Empirical data^1^** |
| C9-O | 1.39 | 1.36 | C9-O-C3 | 115.5 | 119.9 |
| O-C3 | 1.43 | 1.45 | O-C3-N | 112.5 | 113.5 |
| C3-N | 1.47 | 1.43 | C3-N-C2 | 110.7 | 107.9 |
| N-C2 | 1.47 | 1.47 | N-C2-C4 | 107.5 | 110.2 |
| C2-C4 | 1.52 | 1.51 | C2-C4-C9 | 119.8 | 117.7 |
| N-C1 | 1.44 | - | C4-C9-O | 122.9 | 123.1 |
| C9-C4 | 1.40 | 1.39 | C3-N-C1 | 116.1 | - |
